# Concordance Between Indices of Malnutrition: Mid-Upper Arm Circumference V/S Weight for the Height Z Score in Different Age Groups in Karachi, Pakistan

**DOI:** 10.7759/cureus.27387

**Published:** 2022-07-28

**Authors:** Prinka Bai, Heena Rais, Bina Fawad, Sandhya Kumari

**Affiliations:** 1 Pediatric Medicine, Ziauddin University, Karachi, PAK; 2 Community Health Sciences, Ziauddin University, Karachi, PAK; 3 Medicine, Ziauddin University, Karachi, PAK

**Keywords:** malnutrition, wasting, whz, weight-for-height, muac, mid-upper-arm circumference, sam, severe acute malnutrition

## Abstract

Objective

To compare the WHO cut-off of the mid-upper arm circumference (MUAC) with the weight for height z-score (WHZ) in different age groups of children (6 months to 59 months of age) with acute malnutrition in Pakistan.

Methodology

A cross-sectional study was carried out in the pediatric unit of Ziauddin Medical University and Hospital on malnourished children from six to 59 months of age to compare two different indices of malnutrition, MUAC and WHZ. A total of 450 children with WHZ of <-2SD and <-3SD were included in the study after excluding children with failure to thrive due to chronic illness, congenital defects, and immune deficiencies/malabsorption.

Results

The study revealed a significant mean difference in weight, height, and MUAC among the participants (0.030, 0.053, and 0.02). The sensitivity of MUAC at <11.5 cm was highest in the 12-24-month age group with a decline at 24-48 months while specificity was highest at six to 12 months of age, which shows a mixed response.

Conclusion

The result revealed variation in the cut-off value of MUAC in different age groups; the best specificity of MUAC was found at six to 12 months of age and the best sensitivity at 12-24 months of age.

## Introduction

Malnutrition is a common and major health concern of communities in the developing world and malnourished children with complications are at a high risk of mortality [1]. There are around 16 million children below five years of age who are influenced by severe acute malnutrition (SAM) and more than half a million die per year due to severe acute malnutrition [2]. More than 500,000 annual deaths of children can be prevented by the timely management of severely malnourished children [3]. Malnourished children are more likely to struggle with physical and mental growth. Due to decreased immunity, malnourished children are susceptible to recurrent infections and hospital admissions [4]. There is a nine-times higher probability that these children will die than well-nourished children [5]. These children suffer from acute malnutrition, which further harms the growth of the body. In contrast, if children suffering from moderate acute malnutrition (MAM) are not examined on time, it can turn into SAM [6].

Moreover, negligence like lack of knowledge of breastfeeding, lack of a healthy diet rich in macro and micronutrients, poor hygiene, and poverty are some of the many causes of malnutrition.

Thus, the screening of malnutrition in children under five years of age at the community level is extremely important. The two indices used as diagnostic criteria of acute malnutrition are mid-upper arm circumference (MUAC) and weight for height z-score (WHZ) [7]. Children from six months to five years of age with MUAC of <11.5 cm or WHZ < -3 SD are diagnosed as SAM while those with MUAC of <12.5 cm or WHZ between -2 and -3 SD are diagnosed with MAM [8]. While WHZ is considered the gold standard for the diagnosis of malnutrition and MUAC is considered a screening tool at the community level, several children at risk of SAM and MAM are still missed out due to the low sensitivity of MUAC in different age groups [9]. Many studies done in Bangladesh and India suggest increasing the cut-off of MUAC for SAM for effective and efficient screening [10]. Determining the accuracy of the MUAC vs WHZ score in different age groups of Pakistani children will help in improved and effective screening for SAM and MAM [11]. These recommendations will help in developing MUAC cutoff values in different age groups hence preventing avertible mortality and morbidity due to malnutrition [12].

The main objective of this study was to compare the WHO cut-off of MUAC with WHZ in different age groups of children with acute malnutrition in Karachi, Pakistan.

## Materials and methods

A cross-sectional study was carried out at the pediatric unit of Ziauddin Medical University and Hospital. This study was conducted on malnourished children from 6-59 months of age to compare the WHO cut-off of MUAC with WHZ over one year after approval of the institutional review board. The sample size was calculated to be 536 at the 95% confidence interval using Open Epi v 3.0 (www.OpenEpi.com). Children of age six to 59 months with WHZ <-2SD and <-3SD were included in the study and children with failure to thrive due to chronic illness, congenital defects, and immune-deficiencies/malabsorption were excluded from the study. The final sample of the study was 450 after necessary exclusion.

Children of ages six to 59 were recruited after informed consent and divided into four categories: category A (6-12 months), category B (12-24 months), category C (24-48 months), and category D (48-59 months). Ages were confirmed by birth date, calculated in completed months. Additionally, anthropometric measurements, such as weight, MUAC, and height of children, were taken. Standard guidelines were followed while taking anthropometric measurements. WHO provided the non-stretch tape, which was used for the measurement of MUAC (to the nearest 1 mm). Moreover, weight was recorded using a digital scale (weighed to the nearest 0.1 kg) while an infantometer and stadiometer were used to calculate length and height for children <2 years and >2 years, respectively. The data were plotted on the WHO WHZ chart separate for boys and girls, and the results of both indices (MUAC and WHZ) were calculated accordingly. Information about gender, demographic profile, socioeconomic status, and feeding practices was noted. Moreover, the status of education of the mother was determined and the number of children under the age of five was recorded.

The data were analyzed using the SPSS 26 software package (IBM Corp., Armonk, NY). Mean, median, and mode were calculated to identify the normality of data. The chi-square test was utilized to develop the comparison of categorical variables. To compare the continuous variables, a student t-test was used and less than 5% of probability (p-value) was considered a significant value. Additionally, a curve analysis, known as receiver operating characteristics (ROC), was utilized to select the best cut-off value of MUAC in the examination of SAM. Moreover, the area under the curve (AUC) and its standard error were calculated.

## Results

Table 1 shows the characteristics of the research population. Of the total children taken for the study, only 450 met inclusion criteria and were selected. According to the frequency table, there were 242 (53.8%) male and 208 (46.2%) female participants. Results show that out of 450 participants, 192 belonged to the 13-24-month, 156 belonged to the 6-12-month, 65 belonged to the 25-48-month, and the remaining 37 belonged to the 49-60-month age group. Moreover, results have shown that the highly affected group of participants belonged to the middle-middle class. The overall percentage of malnutrition in this study is: SAM was 42.2% and MAM was 57.8%.

**Table 1:** Baseline characteristics SAM: severe acute malnutrition; MAM: moderate acute malnutrition

Characteristics	No. of Children	Percentages
Age in months		
6-12 months	156	34.7
12-24 months	192	42.7
24-48 months	65	14.4
48-60 months	37	8.2
Gender		
Male	242	53.8
Female	208	46.2
Socio Economic Status		
Upper class	57	12.7
Upper middle class	28	6.2
Middle middle class	241	53.6
Lower middle class	124	27.6
Degree of Malnutrition		
SAM	190	42.2
MAM	260	57.8

**Figure 1:**
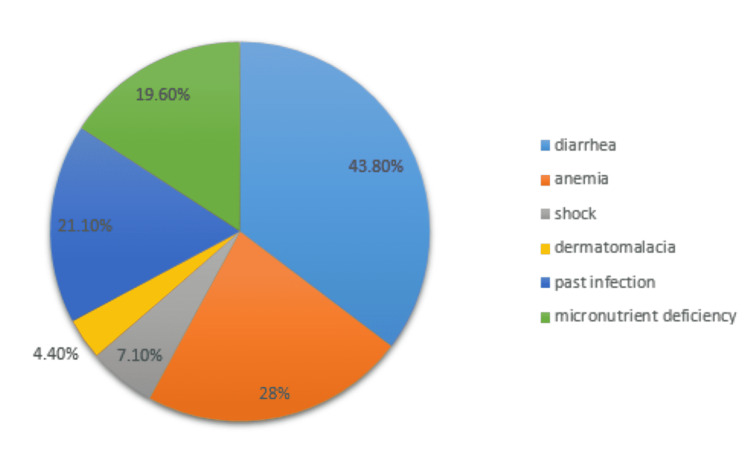
Frequency of complications in malnutrition

Figure 1 demonstrates the co-morbidities that were present in the participants, showing that 43.8% were suffering from diarrhea, 7.1% were suffering from shock, 28% from anemia, 4.4% from dermatomalacia, 21.1% from other micronutrient deficiency while 21.1% had a past medical history of infection. 

Table 2 demonstrates that the sensitivity and specificity of MUAC vary according to age group. The sensitivity and specificity of MUAC < 11.5, i.e., 66% and 92% (age 6-12 months), 88% and 79% (12-24 months), 79% and 80% (24-48 months), and 10% and 81% (48-60 months), respectively. The sensitivity and specificity of MUAC < 12.5, i.e., 39% and 60% % (age 6-12 months), 50% and 55% (12-124 month), 55% and 50% (24-48 months), and 94% and 49% (48-60 months).

**Table 2:** Sensitivity and specificity of MUAC MUAC: mid-upper arm circumference

MUAC	6-12 months		12-24 months		24-48 months		49-60 months	
	Sensitivity	Specificity	Sensitivity	Specificity	Sensitivity	Specificity	Sensitivity	Specificity
10.25	0.878	0.993	0.99	0.926	0.926	0.945	1.000	0.949
10.65	0.865	0.98	0.969	0.919	0.919	0.93	1.000	0.934
10.9	0.859	0.98	0.969	0.915	0.915	0.927	1.000	0.932
11.1	0.66	0.922	0.885	0.791	0.791	0.805	1.000	0.816
11.35	0.654	0.922	0.885	0.787	0.787	0.802	1.000	0.813
11.55	0.609	0.874	0.812	0.76	0.76	0.747	1.000	0.762
11.7	0.609	0.87	0.806	0.76	0.76	0.745	1.000	0.76
11.85	0.603	0.85	0.785	0.748	0.748	0.732	1.000	0.743
11.95	0.603	0.846	0.78	0.748	0.748	0.729	1.000	0.74
12.1	0.391	0.611	0.513	0.55	0.55	0.505	0.946	0.498
12.35	0.391	0.608	0.508	0.55	0.55	0.503	0.946	0.495
12.65	0.353	0.56	0.461	0.508	0.508	0.464	0.946	0.447
12.9	0.353	0.553	0.45	0.508	0.508	0.458	0.946	0.442
13.25	0.179	0.297	0.168	0.322	0.322	0.219	0.649	0.221
13.55	0.071	0.259	0.115	0.252	0.252	0.148	0.649	0.153
13.7	0.071	0.256	0.115	0.248	0.248	0.148	0.649	0.15
13.9	0.071	0.249	0.105	0.248	0.248	0.143	0.649	0.146
14.1	0.058	0.13	0.047	0.147	0.147	0.091	0.459	0.073
14.35	0.058	0.126	0.047	0.143	0.143	0.089	0.432	0.073
14.65	0.019	0.113	0.037	0.112	0.112	0.063	0.378	0.053
14.9	0.019	0.106	0.037	0.105	0.105	0.057	0.324	0.053
15.25	0.019	0.034	0	0.05	0.05	0.016	0.081	0.024
15.75	0	0.034	0	0.039	0.039	0.008	0.081	0.017

**Figure 2:**
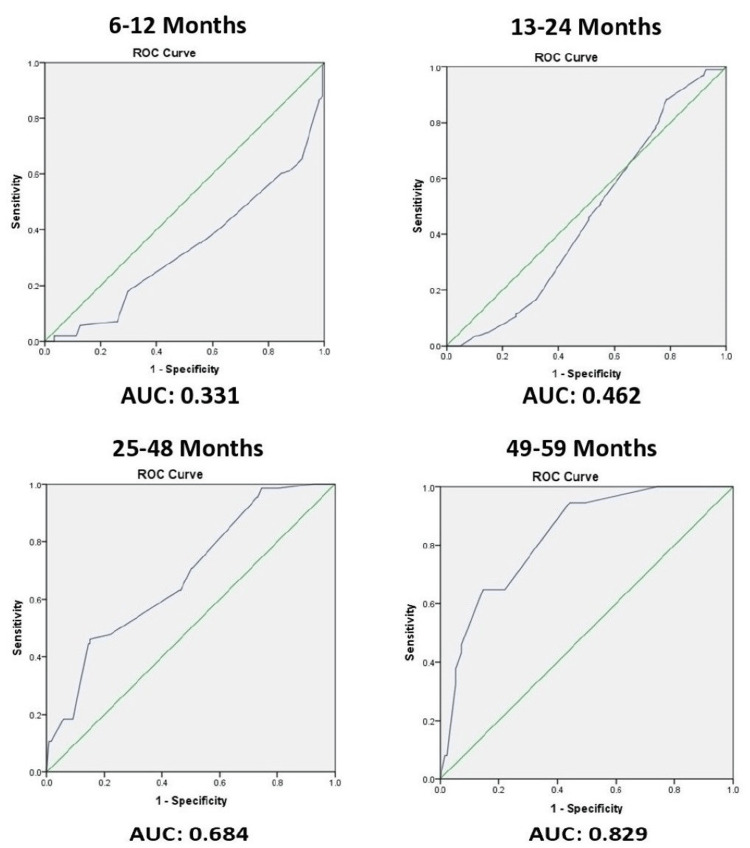
ROC curve showing the sensitivity and specificity of MAUC by AUC MUAC: mid-upper arm circumference; AUC: area under the curve; ROC: receiver operating characteristic

Figure 2 shows the comparison of the ROC curve of MUAC along with the area under the curve (AUC) in different age groups, displaying a high AUC of 0.829 for sensitivity and specificity of MUAC for the 49-60 months age group and the lowest AUC of 0.331 for sensitivity and specificity of MUAC for the 6-12 months age group.

## Discussion

The present study demonstrated that there were several variations found in the diagnosis of SAM through the MUAC and WHZ of the infants. There was a significant mean difference found in weight, height, and MUAC among the participants with a p-value of 0.030, 0.053, and 0.02, respectively. This statement shows that weight, height, and MUAC were all found to help detect the degree of malnutrition present in infants. Furthermore, there was a difference in height/length (cm) in males and females. Our study showed an overall percentage of 42.2% of infants diagnosed with SAM while 57.8% were in the moderate category of malnutrition. The sensitivity of MUAC at <11.5 cm was highest at the age of 12-24 months, with a decline at 24-48 months while specificity was highest at 6-12 months of age, which shows a mixed response.

Marshall et al. found the mean data for height (cm), weight (kg), and MUAC (cm) of infants in their research consistent with our readings (height: 69.5 ± 5.8 vs 79.91 ± 13.51, weight: 7.3 ± 1.3 vs 8.72 ± 2.21, MUAC 13.0 ± 1.1 vs 12.58 ± 1.35) [13]. Our study examined the diagnostic ability of MUAC at 6-12 months, calculating 66% sensitivity and 92% specificity for MUAC <11.5, which was consistent with Marshall et al. Although the opposite was seen in Marshall et al. in the 12-24 months results with high sensitivity (88%) and high specificity (98%). However, these results were conflicting with our readings. One more study by Andreas Chiabi in Cameroon [14] showed similar results as ours, with sensitivity and specificity of MUAC < 11.5 ng.

In our study, the AUC values of 0.331 in 6-12 months and 0.462 in 12-24 month infants were analyzed from the ROC curve of MUAC (cm), which shows the poor diagnostic ability of MUAC. On the other hand, in our study, an AUC value of 0.684 at 24-48 months and 0.829 at 48-60 months were analyzed from the ROC curve of MUAC (cm), showing good prognostic ability with increasing age. On the contrary, this paper found literature with differing findings of AUC from the ROC curve of 0.94 and 0.95 in 6-12 months and 12-24 month infants showing excellent MUAC diagnostic ability [13]. One study from Lahore and one from India, with a value of AUC of 0.78 calculated under a ROC curve of MAUC <11.5 cm at age 6-60 months, showed good predictability [15-16].

Our research has found a positive relationship between the MUAC and Z-scores (p-value: 0.001, r: 0.221), which means that both diagnostic tools can be used to detect SAM. Marshall et al. also studied a strong positive linear relationship between WHZ and MUAC [13]. In another study, it was found that MUAC and WHZ diagnosed 59.9% of 1-6-month infants with SAM [17]. Though there was a profound difference in the age bracket in comparison with this particular research study, this does show that in the early years of birth, the sensitivity and specificity of MUAC and WHZ do have a role to play in the diagnosis of SAM [18].

There was countless literature that gave results that were inconsistent with our research findings. MUAC was stated as a low-sensitivity diagnostic tool with 17.5% (sensitivity) [15]. The credibility was also questioned in a previous study which concluded that MUACs’ low sensitivity properties in increasing age do raise a question of whether they could be a good diagnostic instrument or not [19]. Another research back in 2016 from 27 countries with anthropometric surveys concluded that 52.7% of infants were categorized as suffering from SAM on basis of MUAC and 63.7% of infants based on WHZ scores in the resulting SAM category [6]. Multiple studies agreed on ample concurrence between MUAC and WHZ scores to diagnose SAM [20]. A study in Southern Ethiopia found a fair agreement between MUAC and WHZ scores but results were varying in different age groups [21]. One study in Pakistan found MUAC as a more useful diagnostic tool as compared to WHZ [16]. A study conducted in Sudan gave opposite results, concluding that WHZ was a better method for the diagnosis of SAM [22]. Corresponding to the previous study, a similar statement was made and WHZ was considered better than MUAC [23]. In rural Gambia, an overlap of 59.8% between MUAC and WHZ was found [24]. A recent study done in Cameroon suggested that MUAC <11.5 cm was a better indicator of mortality than WHZ < -3 [14].

The differences in the results could be due to multiple reasons according to the area, environmental conditions, hygiene issues, poverty status, and nutritional status. The present study tried to highlight as much about the diagnostic use of both WHZ scores and MUAC for SAM but conflicting results can be because this is the only study that is specifying MUAC /WHZ according to different age groups. There were comorbidities involved in the current study, which could have affected the preciseness, as it was not known how long they were present and their origin.

This study has a few limitations. For example, due to the coronavirus (COVID) pandemic, a target sample size couldn’t be collected in the given period. Further, this study was conducted in only one center, which might have caused selection biases. More prospective multi-centric studies at the community level need to be conducted for future recommendations regarding WHZ and MAUC.

## Conclusions

This study has shown that wasting, edema, WHZ, and MUAC are independent criteria for screening for malnutrition and are recommended by WHO for decades. The universal value of MUAC < 12.5 cm for the diagnosis of malnutrition is compared by the gold-standard WHZ in this study. The result revealed variation in the cut-off value of MUAC in different age groups; the best specificity of MUAC was found at 6-12 months of age and the best sensitivity at 12-24 months of age. As this was a single-center study, for the generalization of findings at the community level, large multicenter studies need to be done for changing recommendations.
